# K^+^-Dependent Selectivity and External Ca^2+^ Block of Shab K^+^ Channels

**DOI:** 10.1371/journal.pone.0120431

**Published:** 2015-03-23

**Authors:** Elisa Carrillo, Lucero Pacheco, Daniel Balleza, Froylan Gomez-Lagunas

**Affiliations:** Departamento de Fisiología, Universidad Nacional Autónoma de México, DF, México; Cinvestav-IPN, MEXICO

## Abstract

Potassium channels allow the selective flux of K^+^ excluding the smaller, and more abundant in the extracellular solution, Na^+^ ions. Here we show that Shab is a typical K^+^ channel that excludes Na^+^ under bi-ionic, Na_o_/K_i_ or Na_o_/Rb_i_, conditions. However, when internal K^+^ is replaced by Cs^+^ (Na_o_/Cs_i_), stable inward Na^+^ and outward Cs^+^ currents are observed. These currents show that Shab selectivity is not accounted for by protein structural elements alone, as implicit in the snug-fit model of selectivity. Additionally, here we report the block of Shab channels by external Ca^2+^ ions, and compare the effect that internal K^+^ replacement exerts on both Ca^2+^ and TEA block. Our observations indicate that Ca^2+^ blocks the channels at a site located near the external TEA binding site, and that this pore region changes conformation under conditions that allow Na^+^ permeation. In contrast, the latter ion conditions do not significantly affect the binding of quinidine to the pore central cavity. Based on our observations and the structural information derived from the NaK bacterial channel, we hypothesize that Ca^2+^ is probably coordinated by main chain carbonyls of the pore´s first K^+^-binding site.

## Introduction

Potassium channels are proteins that allow the passive and selective flux of K^+^, excluding the smaller, and more abundant in the extracellular solution Na^+^ ions. The structural framework of this selectivity resides in a conserved amino acid signature sequence (TVGYG) [1], which forms the selectivity filter (SF) of the pore [2–4]. Backbone carbonyl oxygen atoms from signature sequence residues point towards the pore lumen, simultaneously coordinating up to two dehydrated K^+^ ions at alternate positions, or binding sites (s1/s3 or s2/s4) [3].

Based on crystal structures, it was proposed that K^+^ is selected over Na^+^ because SF oxygen atoms are positioned at the precise distance and geometry that permits the favorable replacement of the hydration shell of K^+^ (atomic radius = 1.33Ǻ), but not of Na^+^ ions, which have an atomic radius only 0.38 Ǻ smaller than that of K^+^ [2].

The above proposal corresponds to the “snug-fit” model of selectivity [5]. This model does not assign any role to K^+^ ions themselves in the determination of selectivity, and according to it permeation of large ions, such as Cs^+^ (atomic radius = 1.69Ǻ), should also be halted.

Several functional observations do not agree with the snug-fit model. Thus, the proposed SF rigidity stands in contrast with the flexibility of proteins [6,7], and indeed functional evidence indicates that SF is able to undergo sub-Angstrom fluctuations, such as that which accounts for the difference between K^+^ and Na^+^ radius. Some examples comprise experimental observations of the role of K^+^ in the stability of K^+^ conductance [8–13], in particular of the Shaker K^+^ conductance which in the absence of K^+^ collapses in a fully reversible manner [13], demonstrating that the Shaker pore can fluctuate between conducting and, non-inactivated, non-conducting configurations [13,14].

Additionally, other observations demonstrate that in some K^+^ channels, replacement of K^+^ by Na^+^ ions allows the flux of Na^+^, at the moderate membrane potentials at which K^+^ normally flows [15–19]. Moreover, a change in Na^+^ vs. K ^+^ selectivity has been proposed as part of the mechanism of the slow, C-type, inactivation of Shaker [20,21].

In summary, extensive experimental observations regarding stability, gating and selectivity indicate that K^+^-selective pores are flexible structures, although the role of K^+^ ions in these processes continues to be not well understood. A parallel, and to date incompletely characterized phenomenon, is the change in the pharmacological properties of the pore that should accompany K^+^-dependent changes in selectivity, as the latter likely arise from significant changes of pore geometry (e.g., Figure 1 from Hoshi and Armstrong, 2013, [21]).

Herein we report that when internal K^+^ ions are replaced by Cs^+^, a manipulation frequently carried out to eliminate currents through K^+^ channels in cells expressing multiple types of ion channels, stable outward Cs^+^ and inward Na^+^ currents are observed, under bi-ionic Na_o_/Cs_i_ conditions.

The latter shows that selectivity is not accounted for by protein structural elements only, as implicit in the snug-fit model. Additionally, we report the block of Shab channels by external Ca^2+^ ions, and show that ion conditions that undermine selectivity also impair both Ca^2+^ and external TEA block of the pore. Our observations are interpreted within the context of recent structural information acquired with Na^+^-and-K^+^ conducting bacterial channels [22].

## Materials and Methods

### Cell culture and channel expression

Sf9 cells grown at 27°C in Grace’s medium (Gibco) were infected, with a multiplicity of infection of ~10, with a baculovirus containing Shab (dShab 11) K^+^-channel cDNA, as reported [11]. Experiments were conducted 48 h after infection of the cells.

### Electrophysiological recordings

Macroscopic currents were recorded under whole-cell patch-clamp with an Axopatch 1D amplifier (Axon Instruments). Currents were filtered on-line, and sampled at 50 or 100 μsec/point, depending on the experiment, with a Digidata 1322A interface (Axon Instruments). Electrodes were made of borosilicate glass (KIMAX 51) having resistances in the range of 1–1.5 MΩ resistance. The ground electrode was connected to the bath solution through an agar-salt (1M NaCl) bridge. 80% series-resistance compensation was always applied. The holding potential (HP) was -80 mV.

### Solutions

Solutions will be named by their main cation and location across the membrane, e.g., K_i_. Internal solutions contained (in mM): X_i_: 30 XCl, 90 XF, 2 MgCl_2_, 10 EGTA-X, 10 Hepes-X; where X stands for K, Rb, or Cs, as indicated.

External solutions contained (in mM): Na_o_: 145 NaCl, 10 CaCl_2_, 10 Hepes-Na.

NMG_o_: 145 NMG, 10 CaCl_2_, 10 Hepes-H.

10K_o_-NMG_o_: 10 KCl, 135 NMG, 10 CaCl_2_, 10 HEPES-H.

10K_o_-Na_o_: 10KCl, 135 NaCl, 10 CaCl2, 10 HEPES-Na. The pH of all solutions was 7.2.

### Data analysis

Permeability ratios were calculated as: P_x_/P_Y_ = ([Y]_i_/[X]_o_)exp(FV_rev_/RT), where [Y]_i_ & [X]_o_ are the concentrations of Y and X; F, R have their usual meanings, T = 293°K. *t* test was used to evaluate statistical significance.

Junction potentials were estimated following standard procedures [23], accordingly voltage values were corrected off-line as follows: 10K_o_-NMG_o_/Cs_i_: Vm-shift = 6 mV; 10K_o_-Na_o_/Cs_i_: Vm-shift = 8 mV; Na_o_/Cs_i_: Vm-shift = 8 mV. There was less than 1 mV difference between junction potentials of Na_o_/K_i_ and Na_o_/Cs_i_ solutions.

## Results

**Fig 1:**
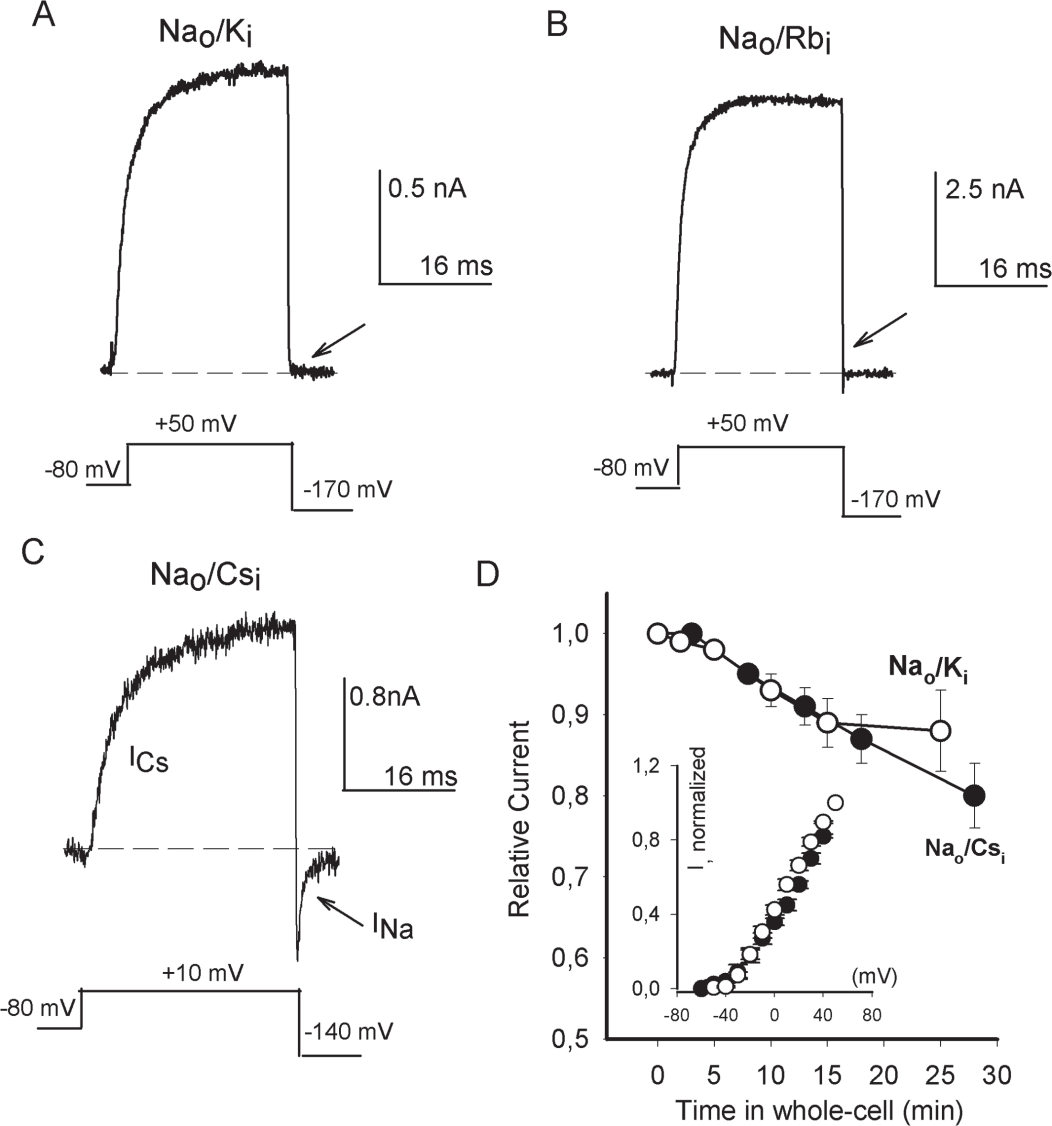
Ion fluxes through Shab channels. (A) I_K_ evoked by a +50mV/30ms depolarization followed by hyperpolarization to -170 mV, as indicated (Na_o_/K_i_ solutions). (B) I_Rb_ evoked as in A (Na_o_/Rb_i_). (C) Outward I_Cs_ evoked by a +10mV/30ms pulse. At pulse end the membrane was hyperpolarized to -140 mV, and an inward I_Na_ was recorded, as indicated (Na_o_/Cs_i_). (D) Relative current vs. time of recording. Relative current = (I(t)/I(0); I(0) is the current amplitude at the beginning of the recording, I(t) is the current remaining at time t, with the cell bathed in either Na_o_/K_i_ (open circles) or Na_o_/Cs_i_ solutions (closed circles), as indicated (see Text). Inset: average normalized outward current recorded in the same solutions (n = 8).

K^+^ channels are defined by their common characteristic of being highly selective for K^+^ over Na^+^ ions. Fig. 1A demonstrates that *Drosophila* Shab channels are typical K^+^ channels. The figure presents a representative K^+^ current (I_K_) evoked by a +50mV/30ms pulse, followed by a strong hyperpolarization to -170 mV, with the cell bathed in bi-ionic Na_o_/K_i_ solutions (see Methods). Note that, despite the huge driving force for Na^+^ ions, there is no appreciably inward current at pulse end (indicated by the arrow). This indicates that P_Na_/P_K_ <0.001 and demonstrates that Shab is a typical K^+^ channel.

To further address Shab selectivity, internal K^+^ (atomic radius = 1.33Ǻ) was replaced by Rb^+^ ions (1.48Ǻ) and channel selectivity (in Na_o_/Rb_i_) was tested as in A. The lack of inward current in Fig. 1B (arrow) shows that the channels exclude Na^+^ as well as they do with K^+^ in the internal solution (P_Na_/P_Rb_ <0.001). This observation agrees with the generalized use of Rb^+^ as a K^+^ substitute, and further endorses Shab as a typical K^+^ channel.

In contrast to the previous observations, Fig. 1C shows that upon replacement of internal K^+^ by the larger Cs^+^ ions (1.69Ǻ) a significant increase in Na^+^ permeability is observed. Thus, with the cell bathed in Na_o_/Cs_i_ solutions, the depolarizing pulse elicits macroscopic outward I_Cs_ which is followed by inward I_Na_, upon membrane hyperpolarization. That is, internal K^+^ replacement by Cs^+^ ions undermines pore selectivity, as assessed by the ability of the channel to conduct Na^+^.

Bathing Shab in Na^+^-containing solutions that lack K^+^ ions irreversibly eliminates the ability of the channels to conduct ions [10–12]). Therefore, we tested the stability of the currents recorded in Na_o_/Cs_i_ solutions. Fig. 1D presents the plot of the relative amplitude of the current as a function of the time of recording (closed circles, Na_o_/Cs_i_). For a reference, the figure also illustrates the stability of I_K_ with standard Na_o_/K_i_ solutions (open circles, data from Ambriz-Rivas et al, 2005 [10]). Note that in Na_o_/Cs_i_ solutions, the stability of the ion conductance is comparable with that observed with physiological [K_i_^+^]. Furthermore, the inset demonstrates that the normalized outward currents, recorded in either of these solutions activate within the same range of potentials. In contrast, we could not obtain stable recordings when K_i_^+^ was replaced by NH_4_^+^ (Na_o_/NH_4i_, not shown).

**Fig 2:**
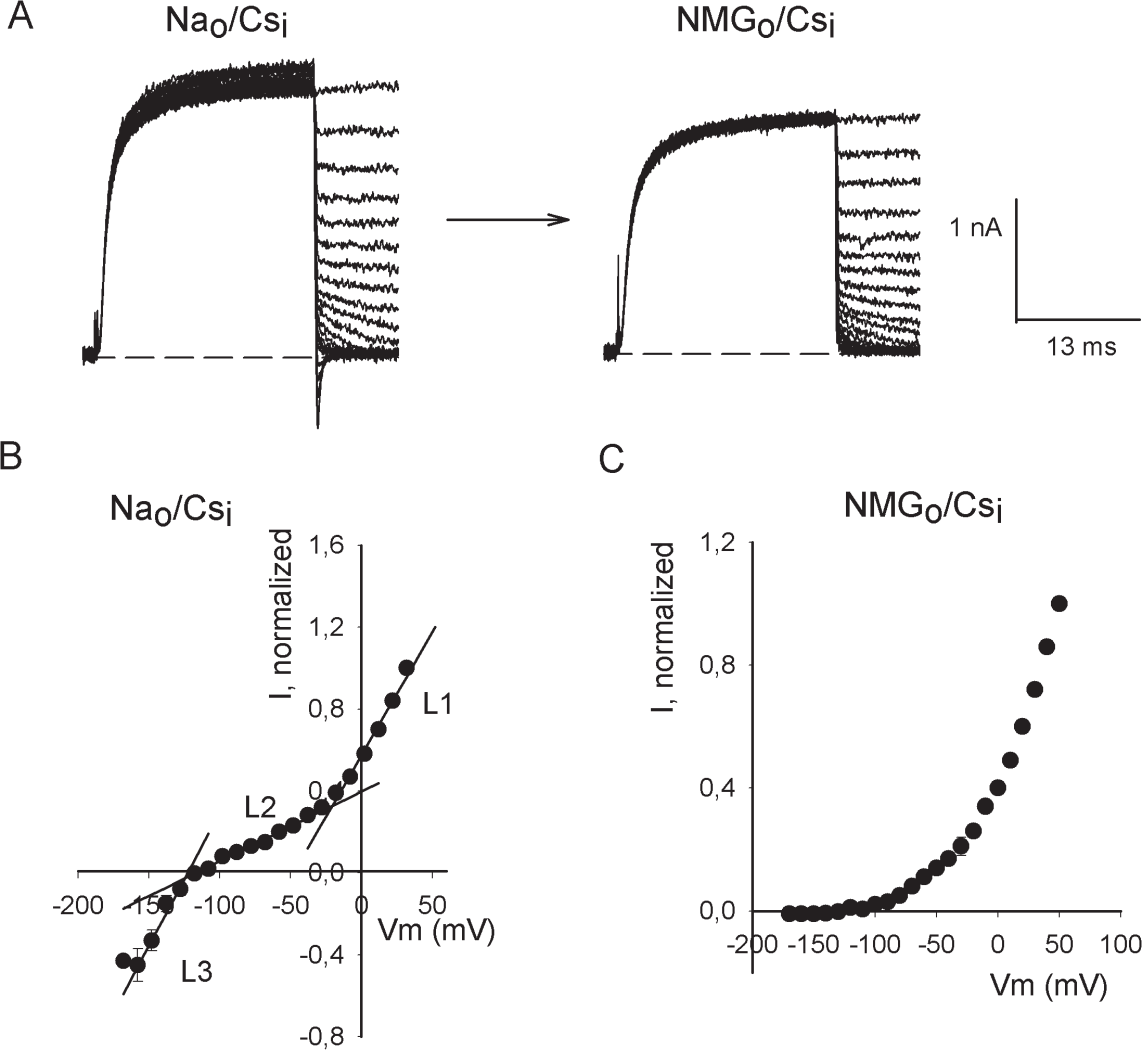
Na^+^ and Cs^+^ currents through Shab channels. (A) Currents recorded applying an instantaneous current vs. voltage protocol (IIV, see Text). Currents were recorded in the same cell in either Na_o_/Cs_i_ (left panel) or NMG_o_/Cs_i_ solutions (right panel). Scale bars are the same for both panels. (B) Average normalized IIV obtained from independent experiments as in the left panel of A (n = 7). L1 to L3 are least squares lines that fit the indicated regions: L1(Vm) = 0.012 Vm + 0.48; r = 0.994; L2(Vm) = 3.4x10^-3^ Vm + 0.37; r = 0.992; L3(Vm) = 0.013 Vm + 1.49; r = 0.990. (C) Average normalized IIV obtained from 4 independent experiments as in the right panel of A.

To further characterize Cs^+^ and Na^+^ permeation we apply an instantaneous I-V protocol (II-V), activating the channels with a +50mV/30ms pulse, and thereafter stepping the voltage from -160 to +40 mV, in 10-mV increments. Fig. 2A left panel illustrates currents recorded with this protocol in a cell bathed in Na_o_/Cs_i_ solutions. Note the inward I_Na_ at negative potentials. The right panel presents the currents obtained in the same cell after the external Na^+^ was replaced by NMG^+^ (indicated by the arrow, NMG_o_/Cs_i_). Note that only the outward I_Cs_ are left, as shown by the average IIV in Fig. 2C. The latter confirms that the inward current observed in Na_o_/Cs_i_ is carried by Na^+^. For the sake of completeness, it is pertinent to mention that we have never observed Li^+^ currents (not shown).

The previous observations are quantified in Fig. 2B and 2C, which present the average normalized II-V obtained with either Na_o_ or NMG_o_ solutions respectively. Regarding the former (Fig. 2B), note that although the overall distribution of the points is not linear, there are three regions of noticeably different constant slopes, as shown by the fitted least squares lines (L1-L3, correlation coefficients≥ 0.99) (see Figure legend). The average of the points of intersection of the voltage axis with L2 of individual experiments yields V_rev_ = -108±3 mV (n = 8), from which a permeability ratio P_Na_/P_Cs_ = 0.01 is obtained.

Concerning the outward I_Cs_, note that in the interval from -120 to -25 mV, I_Cs_ has a relatively small, constant, conductance (L2 slope), whereas positive to -25 mV I_Cs_ presents a ~3.5-fold larger conductance (L1 slope). A small conductance near V_rev_, where the driving force is small, has been explained as possibly arising from single-ion occupancy of the pore [24]. However, in this case with the small conductance interval extending as far as ~80 mV above V_rev_, the former explanation appears improbable. Therefore, it is most likely that, the small conductance indicates that in this region Cs^+^ flux presents a rate limiting energy barrier.

Finally, note that I_Na_ presents a linear variation (L3), within the range of voltages tested, with G_Na_ ~4 G_Cs_ near V_rev_ (L3 slope). Thus, although P_Cs_ >P_Na_, the channel conducts Na^+^ better than Cs^+^ near V_rev_. Finally, note that, at ~ -160 mV, I_Na_ departs from L_3_, suggesting that a region with negative slope would have presented at more negative voltages, similar to that observed with K^+^ present in the external solution (see below and Gomez-Lagunas et al, 2003 [25]).

### Cs^+^ and K^+^ Permeation

**Fig 3:**
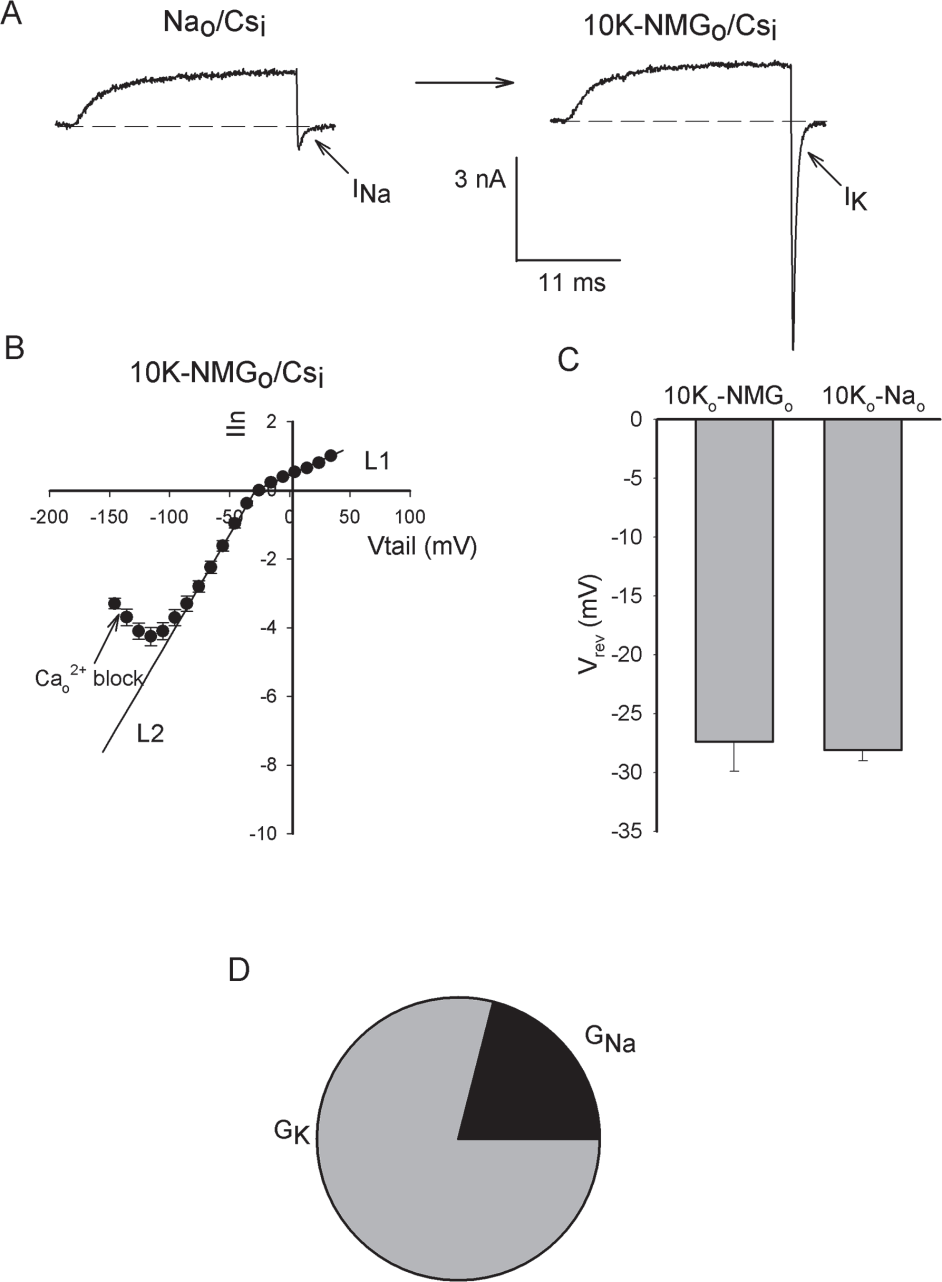
Cs^+^ currents in the presence of external K^+^. (A) Currents recorded in either Na_o_/Cs_i_ (left panel) or 10K_o_-NMG_o_/Cs_i_ solutions (right panel), in the same cell. Currents were evoked by a 0mV/30ms pulse followed by repolarization to -140 mV. (B) Average normalized IIV in 10K_o_-NMG_o_/Cs_i_ solutions (n = 5). The lines are the least squares lines that fit the indicated regions: L1(Vm) = 0.02 Vm + 0.36, r = 0.996; L2(Vm) = 0.06 Vm + 1.37, r = 0.999. (C) Reversal potentials (V_rev_) obtained in 10K_o_-NMG_o_/Cs_i_ vs. 10K_o_-Na_o_/Cs_i_ solutions, as indicated. (D) Relative conductance G_Na_ vs. G_K_ obtained in either Na_o_/Cs_i_ (G_Na_) or 10K_o_-X_o_/Cs_i_ solutions (G_K_); X_o_ represents either Na_o_ or NMG_o_ (see Text).

Fig. 3A compares currents recorded in the same cell in Na_o_/Cs_i_ vs. 10K_o_-NMG_o_/Cs_i_ solutions (see Methods). Note that I_Cs_ has the same amplitude in both solutions (~1.6 nA), suggesting that 10 mM K_o_^+^ does not inhibit outward I_Cs_. On the other hand, note that as expected, at pulse end, the channels clearly conduct K^+^ better than Na^+^.

Fig. 3B presents the instantaneous normalized I-V relationship obtained in 10K_o_-NMG_o_/Cs_i_ solutions. Note that in contrast to the two slopes observed in Fig. 2B, in this case, with 10 mM K_o_^+^, I_Cs_ is fitted by a single slope (L1 slope, see Figure legend). This difference is probably accounted for by the corresponding reversal potentials, which make outward I_Cs_ with 10 mM K_o_^+^ start at a voltage near to that at which the I_Cs_ slope changes in Fig. 2B.

A question of interest was whether this [K_o_^+^] could eliminate Na^+^ permeation. Fig. 3C shows that the reversal potential obtained with NMG ions (10K_o_-NMG_o_/Cs_i_) is basically the same (*P* = 0.617) as the one obtained with Na^+^ ions in the external solution (10K_o_-Na_o_/Cs_i_). The latter demonstrates that 10 mM K_o_^+^ eliminates Na^+^ permeation through the channels, and yields the permeability ratio P_Cs_/P_K_ = 0.17. As a reference, this ratio is about twice as that reported in Shaker channels [26].

Regarding Fig. 3B, note that I_K_ varies linearly from -35 to -75 mV (L2), and as expected G_outward,Cs_<G_inward_,_K_ (for the sake of comparison: G_outward,Cs_/G_inward,K_ = 0.25, although [Cs_i_^+^]/[K_o_^+^] = 12). Similarly, taking the ratio of the minimal squares slopes that fit I_Na_ (Fig. 2B) and I_K_ (Fig. 3B), both obtained with internal Cs^+^, as an indication of the relative conductance of the inward current carried by these ions we obtain G_Na_/G_K_ = 0.21, although [Na_o_^+^]/[K_o_^+^] = 15 (Fig. 3D).

Finally, note that at Vm δ -75 mV the inward current presents a region with a marked negative slope. The latter is the result of a voltage-dependent external Ca^2+^ block of the channels, as shown below.

### Pore block by external Ca^2+^

**Fig 4:**
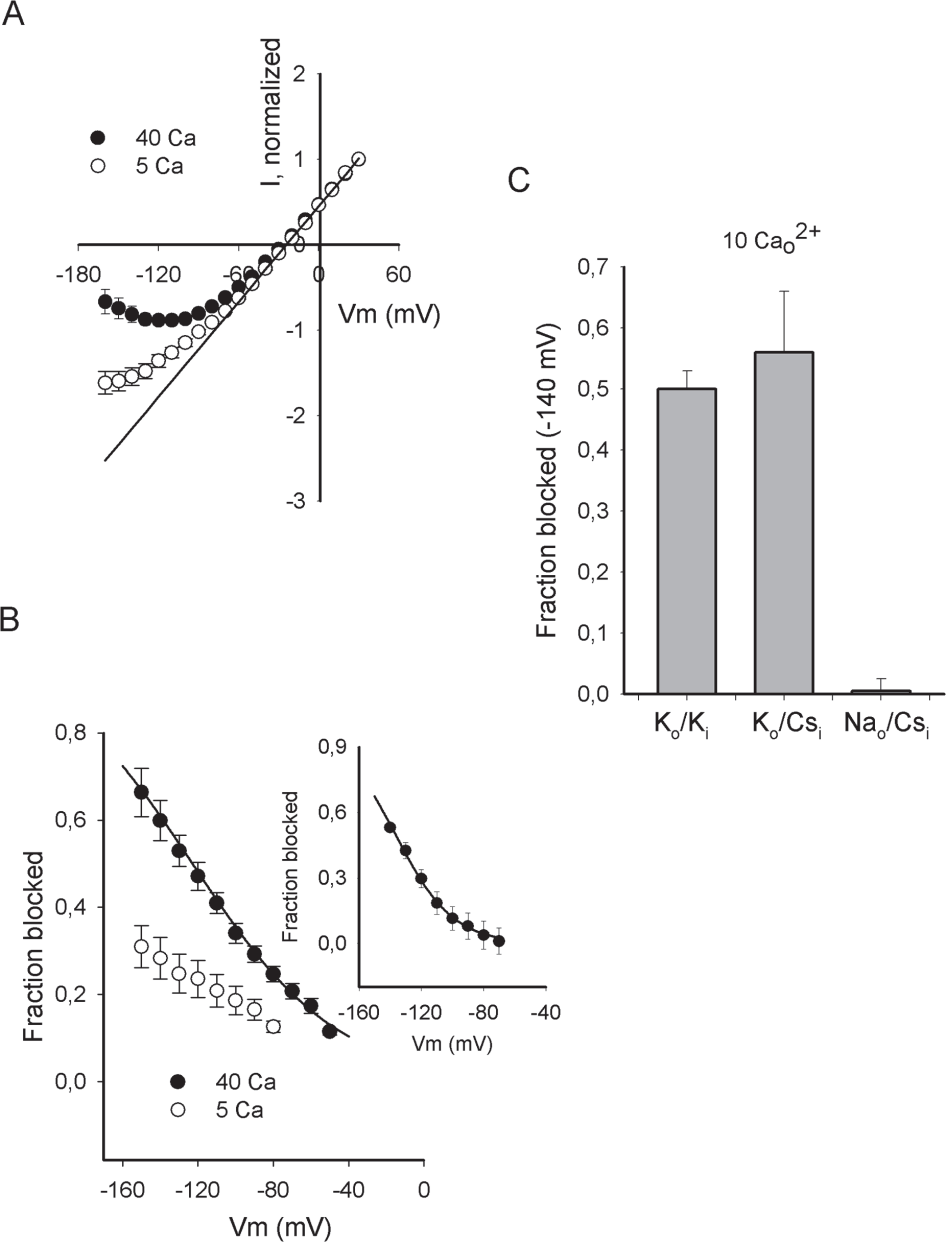
External Ca^2+^ block of inward current. (A) Average normalized IIVs recorded in either 5Ca_o_+30K_o_/K_i_ or 40Ca_o_+30K_o_/K_i_ solutions, as indicated. L is the least squares line fit of the points in the interval from -50 to +30mV. L = 0.45+0.018Vm, r = 0.999. (B) Fraction of channels blocked (fb) by either 5 (open cicles) or 40 mM Ca_o_^2+^ (closed circles) vs Vm. fb was calculated as: fb = 1-(I_K_/I_K,expected_) where I_K_ is the average I_K_ in A, and I_K,expected_ is the value of I_K_ in the absence of block, as obtained from L. The line is the Woodhull equation fit of fb: fb = [Ca_o_]/([Ca_o_]+Kd(0)*exp(-z*δ*F*Vm/RT)), where z = 2 is the valence of Ca^2+^, δ = -0.32 is the electric distance; Kd(0) = 960 mM is the fitted affinity at 0mV; F, R, T have their usual meaning (see Text). The inset presents the fractional block exerted by 10 mM Ca_o_^2+^ in 10K_o_-NMG_o_/Cs_i_ solutions (Fig. 3B). The line is the Woodhull equation fit of fb with δ = -0.68 and Kd(0) = 16736 mM. (C) fractional block exerted by 10 mM Ca_o_^2+^ at -140 mV in the indicated conditions.

Fig. 4A compares instantaneous I-Vs obtained with physiological intracellular K^+^ (K_i_ solution) and 30 mM external K^+^, at two [Ca_o_^2+^]. Note that at Vm τ-50 mV, I_K_ varies in a linear fashion, as demonstrated by the least-squares line that fits the points, but in contrast at hyperpolarized voltages the I-Vs have a clear negative slope, as observed in Fig. 3D.

Departure from linearity at negative voltages clearly depends on the external [Ca^2+^], and the deviation is largest at the higher [Ca_o_^2+^]. This indicates that the negative slope region of the I-Vs is the result of external Ca^2+^ block of the channels, in agreement with previous observations performed in Shaker and plant K^+^ channels [25,27–28].

Ca_o_^2+^ block of Shab is quantified in Fig. 4B, which presents the fractional channel block (fb) as a function of voltage. Block was measured as fb = 1-(I_K_/I_k,expected_), where I_K_ is average I_K_ in Fig. 4A, and I_k,expected_ is the corresponding I_K_ that would have been obtained in the absence of Ca^2+^ block, as evaluated from the least-squares line that fits the points at depolarized voltages (Fig. 4A). The curve is the Woodhull equation fit of the points with 40 mM Ca_o_^2+^ (closed circles), with an electrical distance δ = -0.32 (see Figure legend).

Considering that within the range of voltages tested < 50% of the channels are blocked by 5 mM Ca_o_^2+^ (open circles) the fractional block was not fitted with the Woodhull equation, because the voltage-dependent affinity cannot be properly assessed. Finally, for the sake of clarity the extent of Ca^2+^ block measured with 10 K_o_+10Ca_o_/Cs_i_ solutions (Fig. 3D) is plotted in the inset. The curve is the Woodhull fit of fb with δ = -0.68 (see Discussion).

Interestingly and as previously noted, a comparison of Fig. 2B & Fig. 4 indicates that Ca^2+^ block is basically eliminated in the ionic conditions here reported that undermine pore selectivity, allowing the stable passage of Na^+^. The latter is quantified in Fig. 4C, which compares the fractional Ca^2+^ block ([Ca_o_^2+^] = 10mM) at -140 mV, in ion conditions of interest. Note that the extent of block is similar (*P* = 0.58) in solutions that impede the passage of Na^+^, namely with K^+^ either on both sides of the membrane (30K_o_/K_i_) or in the external solution only (10K_o_/Cs_i_). In contrast, in Na_o_/Cs_i_ solutions Ca^2+^ block is basically eliminated.

The latter suggests that when the channels are bathed in Na_o_/Cs_i_ solutions, the pore conformation changes in a manner that allows Na^+^ permeation and prevents Ca^2+^ block. The addition of 10 mM K_o_^+^ shifts the pore back to its normal Na^+^-excluding conformation, and restores Ca^2+^ block (see Discussion).

### Block by TEA and Quinidine

Finally, considering that the site at which external TEA blocks K^+^ channels has been determined [29], we studied whether the conditions that allow Na^+^ permeation, and eliminate Ca^2+^ block, may also undermine TEA block, as this could suggest the site of Ca^2+^ interaction with the channels.

**Fig 5:**
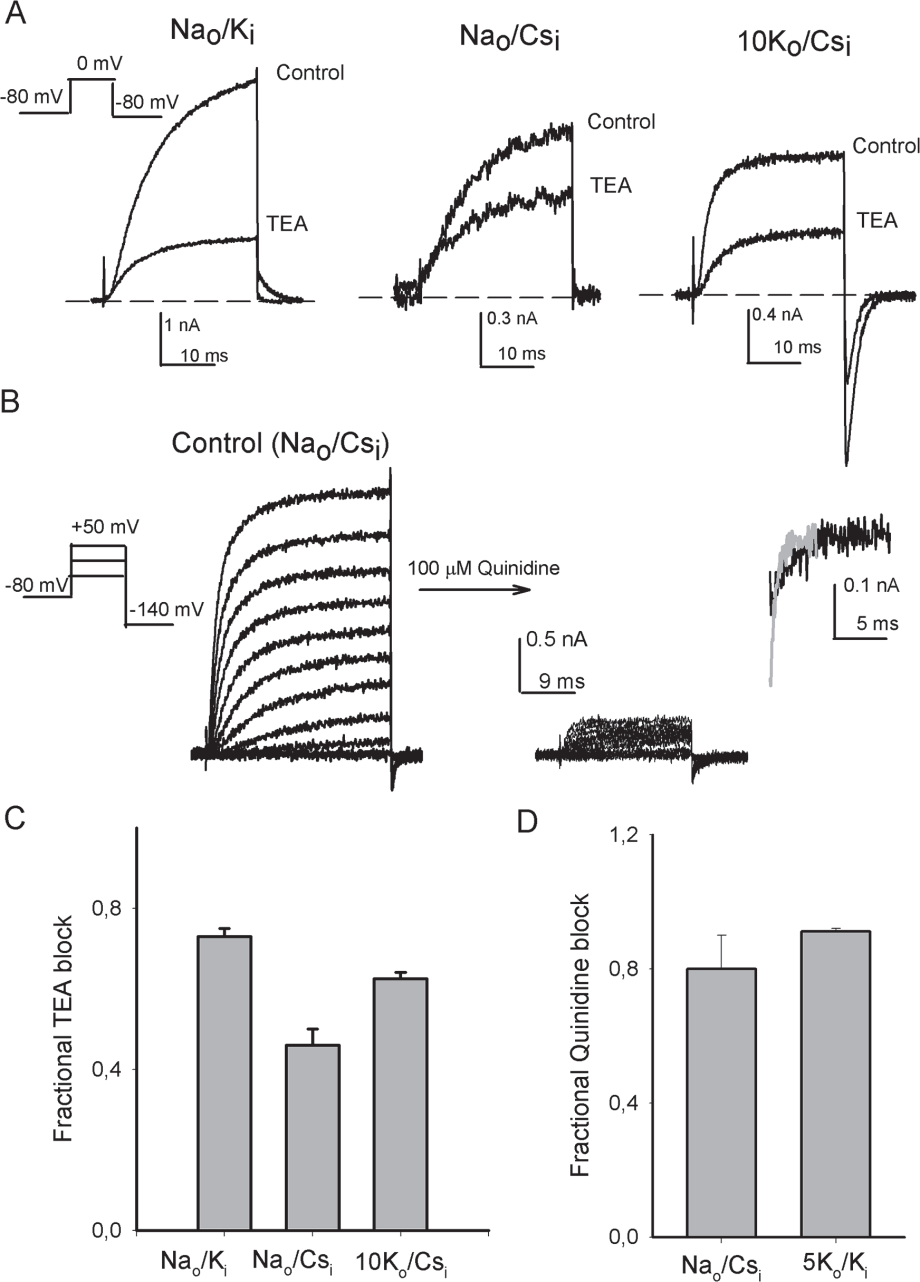
Channel block by TEA and Quinidine, as a function of the recording solutions. (A) Currents illustrating block by 25 mM TEA_o_ at 0 mV, in the indicated conditions (n = 4). Note that traces in Na_o_/Cs_i_ solutions do not present inward Na^+^ current because the repolarization potential is positive to the current reversal potential (B) Currents recorded in Na_o_/Cs_i_ solutions before (left panel) and after the addition of 0.01mM Quinidine (right panel). The inset compares inward Na^+^-tail currents at -140 mV in control (gray trace) control vs. 0.01 mM Quinidine conditions (black trace). (C) Fractional block exerted by 25 mM TEA, as indicated. (D) Fractional block exerted by 0.01mM Quinidine at +50 mV, in the indicated solutions (see Text).

The representative traces in Fig. 5A, and the histogram in Fig. 5C, show that whereas in standard Na_o_/K_i_ solutions 25 mM TEA blocks 73±2% of the channels, in Na_o_/Cs_i_ solutions block is significantly decreased to 46±4% (*P* = 0.009). Additionally, note that addition of 10 mM K_o_^+^ (10K_o_/Cs_i_) restores TEA potency (62±12%, *P* = 0.36). It is worthy to emphasize that currents in Fig. 5A were elicited by a 0 mV depolarization, followed by repolarization to the HP of -80mV (see left panel). The latter explains the lack of inward Na^+^ current upon repolarization in Na_o_/Cs_i_ solutions.

The parallel drop of TEA and Ca^2+^ block, observed under a condition that allows the flux of Na^+^ through the pore, suggests that external Ca^++^ binds in a place located near the TEA_o_ binding site, and that the configuration of this region is changed by the conditions that allow Na^+^ permeation.

Finally, in order to test for possible changes towards the internal side of the pore, we compared the effect of Quinidine on currents recorded in either Na_o_/Cs_i_ or physiological 5K_o_/K_i_ solutions. Quinidine is a compound, that regarding *Drosophila* K^+^ channels is known to specifically block Shab channels, upon binding to the pore central cavity [12].

The traces in Fig. 5B illustrate that addition of 100 μM Quinidine to the external solution blocks ~80% of Shab channels with the cell bathed in Na_o_/Cs_i_ solutions. The inset in the right panel compares the control Na^+^-tail current at -140 mV (gray trace) against the Na^+^- tail current recorded in the presence of Quinidine (black trace). Note that the latter presents a slower time course and an initial hook, as expected from an internal pore blocker that hinders the closing of the activation gate [12].

Finally, Fig. 5D shows that, although slightly bigger, the extent of Quinidine block in physiological 5K_o_/K_i_ solutions is similar to the block exerted in Na_o_/Cs_i_ (*P* = 0.0715) (5K_o_/K_i_ data in Fig. 5D are from Gomez-Lagunas, 2010 [12]). This suggests that pore change(s) that decrease(s) selectivity and external TEA and Ca^2+^ block do not reach the central cavity. Although, further work is needed to understand the parallel ion dependence of TEA and Ca^2+^ block reported in this work. For example, it would be important to determine whether TEA can compete with Ca^2+^ for binding to the pore, and whether mutations known to affect TEA binding also exert en effect on Ca^2+^ block.

## Discussion

Herein we demonstrate that the iso-osmollar replacement of intracellular K^+^ by Cs^+^ ions allows Shab channels to stably conduct both Cs^+^ and Na^+^ ions. This demonstrates that care must be taken in experiments where K^+^ ions are replaced by Cs^+^ ions with the aim of preventing currents trough K^+^ channels. More importantly, our observations show that the presence of K^+^ plays an important role in impeding the flow of Na^+^ under bi-ionic conditions (Na_o_/K_i_), and therefore that K^+^ ions are a cofactor required for maintaining the selectivity, as well as the stability [8–13], of K^+^ pores.

The above indicates that pore selectivity is not fully accounted for by protein structural elements only, as stated in the snug-fit model. Instead, our observations support the alternative Koshland´s induced-fit model [6], according to which ion binding sites are not rigidly positioned, and selectivity depends on the balance between the ions hydration energy and the strain energy required by the protein to properly coordinate the ions [6,30]. As a result, ions compete for available binding sites [16], and are selected according to the balance of their corresponding energies.

Our observations agree with former observations [15–19], in particular with experiments showing that, upon K^+^ replacement by Na^+^ ions, Kv2.1 channels allow the passage of Na^+^ [16]. On the other hand, in Shab, the Drosophila homolog of Kv2.1, the same ion substitution deranges the pore in a manner that a fast and irreversible collapse of the ion conductance takes place [10–12].

For the sake of completeness, it should be noted that despite the wealth of work that has been devoted to determine the mechanism of selectivity controversy still exist regarding the mechanism by which ions are selected, with the snug fit model still being favored by some authors (e.g., see Noskov & Roux (2006) [6], and Derebe et al (2011) [31]).

Although under standard bi-ionic conditions (Na_o_/K_i_) Shab does not conduct Na^+^, it is nonetheless pertinent to discuss our results regarding Na^+^ conduction within the framework of observations recently obtained in a Na^+^ and K^+^ conducting bacterial channel (NaK), which presents a pore architecture similar to that of K^+^ channels [2,22].

The NaK pore presents only two ion binding sites, which otherwise are chemically identical to the innermost K^+^ sites of KcsA (s3, s4), and yet NaK lacks K^+^-selectivity [22]. Amino-acid substitutions that produced functional NaK pores endowed with a variable number of binding sites, led to the interesting proposal that K^+^ selectivity requires the presence of 4 in-line K^+^ binding sites, as observed in KcsA [3,31].

Our observations show that in the case of Shab the iso-osmollar substitution of K_i_^+^ by Cs^+^ changes the pore geometry, in such a way that it becomes able to conduct Na^+^. Based on the results obtained with the NaK channel [31], we hypothesize that pore occupancy by Cs^+^ somehow reduces the effective number of ion binding sites, probably by inducing a small change in the geometry of coordinating carbonyls that point to the pore lumen, probably similar to the one thought to occur in the outermost site (s1) of Shaker channels during C-type inactivation [20,21]. In support of the latter possibility, we observed that bathing the channels in Na_o_/Cs_i_ solutions brings a change in the conduction pathway that affects the extracellular side of the pore, as deduced by the decreased potency of block by external TEA and Ca^2+^ ions, but not of quinidine.

In Kv2.1 channels substitution of K^+^ by Na^+^ renders the channel resistant to TEA_o_. The latter involves the displacement of lysine at position 382, in the external vestibule of the pore, which hinders TEA binding [32,33]. Shab presents a threonine at the equivalent position, and further work is needed to determine whether a phenomenon similar to the one in Kv2.1 underlines the reduced potency of TEA block observed in Na_o_/Cs_i_.

More important for the present discussion, the parallel elimination of Ca^2+^ block observed under the previously mentioned conditions suggests that Ca_o_^2+^ binds to the channels near the TEA_o_ binding site, at the pore entry [34], notwithstanding the obtained values of δ, because it is known that δ does not indicate of a physical distance, and that instead its value is the result of the interaction between blocking and permeant ions [35,36]. The latter underlies the different values of δ obtained using either 30K_o_/K_i_ (δ = -0.32) or K_o_/Cs_i_ solutions (δ = -0.65).

Several proteins bind Ca^2+^ with oxygen atoms provided by serine, threonine, or carboxyl groups of either aspartate or glutamate side chains, as observed for example in calmodulin [6]. On the other hand, recent crystallographic images of cation channels show that Ca^2+^ can also bind to main chain carbonyl groups that point to the pore lumen [37].

Therefore, based on the one hand on the observed parallel decrease of TEA_o_ and Ca_o_^2+^ block of Shab, under conditions that allow the passage of Na^+^, and on the other hand based on crystal structures exhibiting Ca^2+^ ions bound to selectivity filter carbonyls [37], we hypothesize that Ca^2+^ blocks the *Drosophila* Shab, and other K^+^ channels like Shaker [25], by binding at the pore entry, probably above the first K^+^ binding site (s1).

In this scenario, the loss of selectivity and Ca^2+^ block, observed in Na_o_/Cs_i_ solutions, could probably arise from a change in the geometry of s1. The presence of 10 mM K_o_^+^ inhibits Na^+^ permeation and restores Ca_o_^2+^ block probably by impeding the change of geometry of this site. In agreement with this hypothesis, the observations regarding Quinidine block suggest that the central cavity remains unchanged by conditions that allow Na^+^ permeation. Finally, for the sake of completeness, it must be mentioned that the absence of Ca^2+^ ions at the pore entry noticed in crystal structures of K^+^ channels [37], might be the result of the voltage dependence of Ca_o_^2+^ block which requires negative voltages (≤-70 mV), absent in crystal structures, to develop.
